# The impact of dusk phenomenon on total glucose exposure in Chinese people with type 2 diabetes

**DOI:** 10.1097/MD.0000000000025298

**Published:** 2021-04-02

**Authors:** Yuxin Huang, Yuanyuan Xu, Jieyuzhen Qiu, Cuiping Jiang, Wen Tan, Xiaoming Tao, Qin Gu, Jiao Sun

**Affiliations:** Department of Endocrinology, Huadong Hospital Affiliated to Fudan University, Shanghai, PR China.

**Keywords:** Chinese people, continuous glucose monitoring, dawn phenomenon, dusk phenomenon, type 2 diabetes

## Abstract

This study was aimed at assessing the impact of the dusk phenomenon on the total glucose exposure in Chinese people with type 2 diabetes.

A total of 380 type 2 diabetes who received a retrospective continuous glucose monitoring system (CGMs) for 72 hours were enrolled in our study, 32 of them failed in CGMs. The patients were first divided into 2 groups: dusk phenomenon (n = 95) and non dusk phenomenon group (n = 253). The magnitude of the dusk phenomenon (δDusk) was quantified by pre-dinner glucose minus post-lunch 2 hours glucose. A persistent δDusk ≥ 0 or a once only δDusk < 0 can be diagnosed with the dusk phenomenon. The participants were secondarily matched for the post-lunch 2 hours glucose to assess the impact of the dusk phenomenon on the overall glucose exposure. The impact of the dusk phenomenon was assessed on high-performance liquid chromatography assay (HbA1c) and 24-hour mean glucose.

There were 95 of 348 (27.3%) participants with the dusk phenomenon in the overall population, and the median of δDusk level was –0.8 (–1.8, 0.2) mmol/L. The median of glucose differences between the 2 paired groups were 0.4 (–0.4, 1.0)% for HbA_1c_, 0.9 (0.2, 1.4) mmol/L for 24 hours mean glucose. The correlation analysis showed no relationship between the magnitude of dawn phenomenon and the dusk phenomenon (*r* = 0.052, *P* = .472).

The incidence of dusk phenomenon is about 27.3% in people with type 2 diabetes. The impacts of dusk phenomenon on HbA1c and 24-hour mean glucose were about 0.4% and 0.9 mmol/L and the dusk phenomenon was not related with the dawn phenomenon.

## 1 Introduction

Glucose fluctuation, also known as glucose variability or glycemic excursion, refers to the unstable state of the change of blood glucose level between its peak and valley. Glucose fluctuations were reported to be related to oxidative stress, endothelial dysfunction, and inflammation, factors traditionally associated with the pathogenesis of cardiovascular disease.^[1]^ In the past 10 years, more and more endocrinologists have begun to pay attention to glucose fluctuations. A large number of studies have confirmed that glucose fluctuations are harmful to people with diabetes, including diabetic peripheral neuropathy,^[2]^ diabetic retinopathy,^[3]^ urinary albumin excretion,^[4]^ increased carotid artery intima-media thickness,^[5]^ coronary artery spasm,^[6]^ coronary artery disease,^[7]^ longer hospitalization, and increased short-term and long-term mortality.^[8]^ Furthermore, glucose excursion and postprandial glucose were higher among East Asian patients with diabetes than Caucasian patients.^[9]^ Therefore, we should pay more attention to glucose fluctuation in Chinese people with diabetes.

There are 2 special mechanisms involved in glucose fluctuation: the “dawn phenomenon” and the “dusk phenomenon.” The term “dawn phenomenon” was first introduced by Schmidt et al^[10]^ in 1981 and was used to describe fasting hyperglycemia or a spontaneous rise in insulin requirements during the early morning, occurring in the absence of nocturnal hypoglycemia. It is very common in both people with type 1 diabetes and with type 2 diabetes.^[11]^ The incidence of dawn phenomenon was similar among patients with different oral antidiabetic drugs, even when given as combined therapies.^[12,13]^ By using continuous glucose monitoring system (CGMs), dawn phenomenon could be determined by the difference between nocturnal nadir and pre-breakfast glucose levels.^[11]^

Oppositely, the dusk phenomenon is not as well-known as the dawn phenomenon. The term “dusk phenomenon” only exists in concepts and very few studies have dealt with this topic.^[14,15]^ It was defined as spontaneous and transient pre-dinner hyperglycemia in people with diabetes as reported.^[14]^ A consecutive dusk glucose difference >0 or a once only dusk glucose difference <0 could be diagnosed as the dusk phenomenon, while the dusk phenomenon could be excluded when a consecutive dusk glucose difference <0 or a once only dusk glucose difference >0 was found.^[15]^ The dusk phenomenon was proved to be an objective existence in both people with type 1 diabetes and with type 2 diabetes. In addition, the blood glucose level remained rising from pre-dinner to pre-bed in people with the dusk phenomenon.^[14]^

In this study, we investigated: assess the impact of the dusk phenomenon on the total glucose exposure in Chinese people with type 2 diabetes; whether the dusk phenomenon is related to the dawn phenomenon. This might incite health care providers to devise therapeutic strategies to avoid this phenomenon in an attempt to achieve better overall glycemic control in people with type 2 diabetes.

## 2 Materials and methods

### 2.1 Study populations

Three hundred eighty people with type 2 diabetes (diagnosed according to the 1999 World Health Organization criteria), diet only or stable treatment with metformin for at least 3 months, aged ≥18 years, with body mass index (BMI) ≥18.5 kg/m^2^, and with a high-performance liquid chromatography assay (HbA_1c_) < 9.0% were enrolled in our study. All participants were recruited from Department of Endocrinology, Huadong Hospital affiliated to Fudan University, Shanghai, China. The exclusion criteria included the following: current diabetic ketoacidosis or hyperosmolar coma; current hypoglycemia or suspected hypoglycemia; current cardiovascular disease or other serious disease; severe hepatic insufficiency or renal insufficiency; confirmed or suspected type 1 diabetes; use of unknown combination drugs; and poor compliance. The study protocol was approved by the Ethics Committee of Huadong Hospital (2018K065). All procedures involving human participants were performed in accordance with the ethical standards of the Helsinki declaration. Informed consent was obtained from all individuals included in the study.

### 2.2 Clinical investigations, laboratory examinations

Following a 10 hour night fast, anthropometric and blood samples were collected. Laboratory measurements of plasma glucose, insulin, C-peptide, HbA_1c_, and lipid profiles were performed in a central laboratory of Huadong Hospital (221 yananxi road, Shanghai, 200040, PR China). BMI was calculated as one's weight (kg) divided by the square of one's height (m). Insulin sensitivity was calculated as the homeostatic model assessment of insulin resistance index (HOMA-IR), and baseline insulin secretion was calculated as the homeostatic model assessment of β-cell function (HOMA-β) using the HOMA calculator (Headington, Oxford, UK) (http://www.dtu.ox.ac.uk).

### 2.3 Medical nutrition therapy

All participants were instructed to maintain their recommended medication. All participants received individualized medical nutrition therapy from registered nutritionists. Energy intake was assumed to be equal to energy expenditure, and carbohydrates provided 50% of the total daily energy intake. Breakfast, lunch, and dinner were calorically divided as 1:2:2. The three daily meals were required to be ingested between 7:00 and 7:30, 11:00 and 11:30, and 17:00 and 17:30, respectively. Participants were not allowed to consume any snacks between meals.

### 2.4 Continuous glucose monitoring and magnitude of the dusk phenomenon

All participants were evaluated using a retrospective CGMs (MiniMed system, Medtronic Inc., California) for 72 hours. Participants were obliged to input their capillary blood glucose at least 4 times a day for adjusting the CGMs. The sensor of CGMs was installed on day 0 and removed on day 3.

The magnitude of the dawn phenomenon (δDawn) was quantified by pre-breakfast glucose minus nocturnal nadir glucose, and the threshold of δDawn was determined to be 20 mg/dL (1.11 mmol/L) as reported.^[12,16]^ The magnitude of the dusk phenomenon (δDusk) was quantified by pre-dinner glucose minus post-lunch 2 hours glucose. A persistent δDusk ≥ 0 or a once only δDusk < 0 can be diagnosed with the dusk phenomenon as reported.^[15]^ Then the data provided from CGMs were obtained during day 1 and day 2 to avoid any interference due to installation and removal of the sensor. Furthermore, all glucose values recorded on day 1 and day 2 were averaged in order to avoid bias.

### 2.5 Grouping

**Figure 1:**
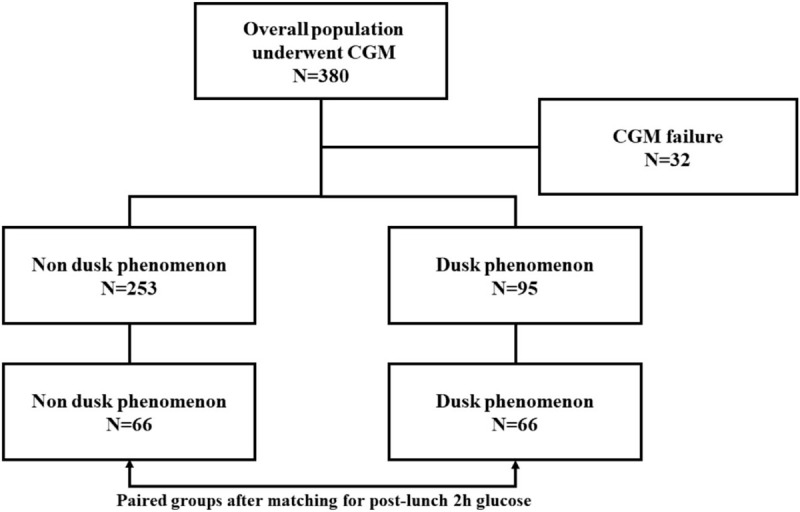
Participants disposition and study protocol. Participants of the total investigated population were firstly divided into 2 groups according to whether they exhibited or not the dusk phenomenon. All separated participants were secondarily matched for the post-lunch 2 hours glucose that was taken as reference for the calculation of the dusk glucose increment.

Participants of the total investigated population were firstly divided into 2 groups according to whether they exhibited or not the dusk phenomenon. In order to assess the impact of the dusk phenomenon on the overall glucose exposure, all separated participants were secondarily matched for the post-lunch 2 hours glucose that was taken as reference for the calculation of the dusk glucose increment (see Fig. 1). Similar to the method proposed by Monnier et al,^[12]^ the impact of the dusk phenomenon on glucose profiles was then quantified by comparing the mean differences in the averaged glucose and HbA_1c_ levels between the paired groups who were matched for post-lunch 2 hours glucose and separated according to the presence or absence of the dusk phenomenon.

### 2.6 Statistical analysis

Case-control matching and statistical analysis were performed with SPSS 23.0 software (SPSS Inc., Chicago, IL). Normally distributed data are presented as mean ± SD, and non-normally distributed data are presented as median (interquartile range, 25th–75th percentile). Clinical characteristics with normal distribution were analyzed using *t* test, while those with non-normal distribution were compared by the Kruskal–Wallis test. Categorical variables were expressed as numbers (%) and analyzed by using the chi-squared test. Pearson correlation coefficients were calculated to assess the strength of correlations between δDawn and δDusk. Logistic regression was used to examine the relationship between the presence/absence of dusk phenomenon and independent variables. All *P* values were 2-tailed and *P* values <.05 were considered significant. Artworks were created by Microsoft PowerPoint 2013 (Microsoft Inc., Redmond, Washington) and GraphPad Prism 5 (GraphPad Software, San Diego, California).

## 3 Results

### 3.1 Clinical characteristics and glucose profiles in the overall population

A total of 380 participants enrolled in our study and underwent CGMs. Thirty two of them failed in CGMs, including suspicious hypoglycemia, incomplete glucose data, device error, etc (see Fig. 1). The clinical characteristics of the rest 348 participants are summarized in Table 1. The mean age was 63.1 ± 11.5 year, BMI was 25.1 ± 3.2 kg/m^2^, HbA_1c_ was 7.0 ± 0.8%, 24 hours mean glucose was 7.5 ± 1.2 mmol/L, HOMA-IR was 3.4 ± 1.7, and the median of HOMA-β was 60.0 (40.6, 84.1).

**Table 1:** Main clinical characteristics of patients in the overall population and in the different groups after selection for presence/absence of the dusk phenomenon and after matching for post-lunch 2 hours glucose.

	Overall	Non DP	DP	Impact of DP	*P* value
Number	348	66	66		
Male/female	190/158	39/27	33/33		.382
Age, y	63.1 ± 11.5	62.2 ± 11.7	63.1 ± 10.0		.638
Body mass index, kg/m^2^	25.1 ± 3.2	25.5 ± 3.0	25.3 ± 2.9		.716
HOMA-IR	3.4 ± 1.7	3.1 ± 1.7	3.3 ± 1.3		.300
HOMA-β	60.0 (40.6, 84.1)	63.6 (44.8, 91.2)	48.6 (33.0, 75.2)		.009
Mean glucose value, mmol/L					
Nocturnal nadir	5.8 ± 1.2	5.4 ± 0.8	6.2 ± 1.4	0.9 (–0.3, 1.7)	<.0001
Pre-breakfast	7.1 ± 1.4	6.7 ± 1.2	7.5 ± 1.3	0.8 (0, 1.8)	.001
Post-breakfast 2 hour	8.8 ± 2.2	8.1 ± 1.9	9.2 ± 2.0	1.2 (–0.6, 2.8)	.003
Pre-lunch	7.5 ± 2.1	6.8 ± 1.6	7.7 ± 2.1	0.6 (–0.8, 2.2)	.004
Post-lunch 2 hour	8.2 ± 1.9	7.7 ± 1.3	7.7 ± 1.3	0	1.000
Pre-dinner	7.3 ± 1.8	6.3 ± 1.2	8.8 ± 1.8	2.1 (1.2, 3.3)	<.0001
Post-dinner 2 hour	8.4 ± 2.0	7.8 ± 1.7	9.0 ± 2.0	1.2 (–0.1, 2.8)	<.0001
HbA_1c_ (%)	7.0 ± 0.8	6.7 ± 0.8	7.1 ± 0.8	0.4 (–0.4, 1.0)	.004
24 hours mean glucose, mmol/L	7.5 ± 1.2	7.1 ± 0.9	8.1 ± 1.0	0.9 (0.2, 1.4)	<.0001
δDawn, mmol/L	1.0 (0.3, 1.8)	1.0 (0.3, 1.7)	1.2 (0.4, 1.8)		.935
δDusk, mmol/L	–0.8 (–1.8, 0.2)	–0.9 (–1.7, –0.4)	0.7 (0.3, 1.5)		<.0001

Data are means ± SD, median (interquartile range) or number (percentage). The impact of the dusk phenomenon was assessed by calculating the differences between HbA_1c_ levels and glucose profiles in different groups of patients with/without a dusk phenomenon and matched for post-lunch 2 hours glucose. δDawn = pre-breakfast glucose minus nocturnal nadir glucose, δDusk = pre-dinner glucose minus post-lunch 2 hours glucose, DP = dusk phenomenon, HOMA-β = homeostasis model assessment-β cell function, HOMA-IR = homeostasis model assessment-insulin resistance. *P* values indicate the statistical significance between non DP and DP.

There were 95 of 348 (27.3%) participants with the dusk phenomenon in the overall population, and the median of δDusk level was –0.8 (–1.8, 0.2) mmol/L. Even in participants with HbA_1c_ level <7%, there were still 43 of 182 (23.6%) participants with the dusk phenomenon. There was no significant difference in the incidence of the dusk phenomenon between those on diet only and those on metformin (*P* = .369).

### 3.2 Clinical characteristics and glucose profiles in the different groups

The included 348 participants were firstly separated into 2 groups according to whether they exhibited (N = 95) or not (N = 253) the dusk phenomenon. All separated participants were secondarily matched for the post-lunch 2 hours glucose: 66 without the dusk phenomenon and 66 with the dusk phenomenon, see Fig. 1. Age, sex, BMI, and HOMA-IR did not differ significantly between the 2 groups (all *P* > .05). Participants with the dusk phenomenon had lower HOMA-β level (48.6 [33.0, 75.2] vs 63.6 [44.8, 91.2], *P* = .009) than those without dusk phenomenon.

**Figure 2:**
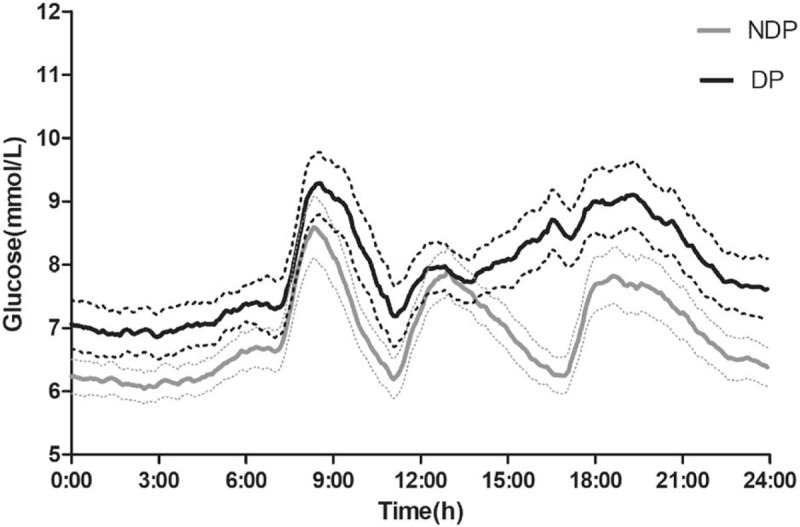
Continuous glucose profiles of people with dusk phenomenon and people without dusk phenomenon. Black solid line = people with dusk phenomenon (DP); black dotted line = 95% confidence interval for people with dusk phenomenon (DP); grey solid line = people without dusk phenomenon (NDP). Grey dotted line = 95% confidence interval for people without dusk phenomenon (NDP).

The 24 hours glucose profiles, magnitude of the dawn phenomenon (δDawn), and magnitude of the dusk phenomenon (δDusk) in the 2 groups are summarized in Table 1 and Fig. 2. Participants with the dusk phenomenon presented with higher nocturnal nadir glucose (*P* < .0001), higher pre-breakfast glucose (*P* = .001), higher post-breakfast 2 hours glucose (*P* = .003), higher pre-lunch glucose (*P* = .004), higher pre-dinner glucose (*P* < .0001), and higher post-dinner 2 hours glucose (*P* < .0001) than those without the dusk phenomenon. Participants with dusk phenomenon also presented with higher HbA_1c_ level (7.1 ± 0.8% vs 6.7 ± 0.8%, *P* = .004) and higher 24 hours mean glucose level (8.1 ± 1.0 mmol/L vs 7.1 ± 0.9 mmol/L, *P* < .0001).

### 3.3 Impact of the dusk phenomenon on glucose profiles

The impact of the dusk phenomenon was assessed by calculating the differences between HbA_1c_ levels and glucose profiles in different groups of participants with/without a dusk phenomenon and matched for post-lunch 2 hours glucose, see Table 1. The median of glucose differences between the 2 paired groups were 0.4 (–0.4, 1.0)% for HbA_1c_, 0.9 (0.2, 1.4) mmol/L for 24 hours mean glucose, 0.9 (–0.3, 1.7) mmol/L for nocturnal nadir, 0.8 (0, 1.8) mmol/L for pre-breakfast glucose, 1.2 (–0.6, 2.8) mmol/L for post-breakfast 2 hours glucose, 0.6 (–0.8, 2.2) mmol/L for pre-lunch glucose, 2.1 (1.2, 3.3) mmol/L for pre-dinner glucose, and 1.2 (–0.1, 2.8) mmol/L for post-dinner 2 hours glucose.

### 3.4 Correlation and regression analysis

The correlation analysis showed no relationship between δDawn and δDusk (*r* = 0.052, *P* = .472). Binary logistic regression was calculated to evaluate the relationship between the presence of dusk phenomenon and the following independent variables: sex, age, BMI, HbA_1c_, HOMA-IR, HOMA-β, and δDawn. Regression analysis showed that only HOMA-β (odds ratio [OR] = 0.989, 95% confidence interval [CI]: 0.980–0.998, *P* = .017) was significant independent correlates with the dusk phenomenon.

## 4 Discussion

The concept of dusk phenomenon is both familiar and unfamiliar to endocrinologists. Although there has been a lack of definition for a long time, the dusk phenomenon is often encountered in clinical practice. Scheiner and Boyer^[17]^ conducted a study in 2005 that involved 322 patients with type 1 diabetes. Researchers had these patients fast at different times during the day and then adjusted the basal insulin until their glucose levels were within the glycemic target. Interestingly, they could not set just 1 or 2 basal insulin rates and achieve most patient's glycemic targets. The basal insulin had to be adjusted to several different basal rates throughout the day. Most patients presented 2 peaks of insulin requirement: one in the morning and the other after dinner time. In patients treated with continuous subcutaneous insulin infusion, the basal insulin was recommended to be programmed to increase in the early morning (for the dawn phenomenon), decrease in the afternoon, and then increase in dinner time (probably for the dusk phenomenon).^[18]^ In addition, a study of 434 healthy Chinese people with normal glucose regulation showed that post-dinner glucose were the highest among 3 meals.^[19]^ These above studies may indicate the existence of dusk phenomenon.

The direct evidence and diagnostic criteria of the dusk phenomenon came from a series of studies.^[14,15]^ They enrolled diabetic patients treating with continuous subcutaneous insulin infusions to observe the dusk phenomenon. Because giving the pre-prandial insulin via a continuous insulin pump could partly eliminate the influence of increased glucose, the abnormally increased glucose in the patients with the dusk phenomenon will then be exposed to show higher glucose levels pre-dinner compared with post-lunch 2 hours. According to their studies, a consecutive δDusk >0 or a once only δDusk <0 could be diagnosed as the dusk phenomenon.^[15]^ However, the HbA_1c_ level was very high in their study (11.1 ± 1.52%). Although insulin pump was used, unstable glycemic control could affect the observation of the dusk phenomenon. Thus in our study, we enrolled type 2 diabetes with diet only or stable treatment of metformin for at least 3 months and with HbA_1c_ < 9.0% in order to provide an ideal platform for the observation of the dusk phenomenon. In our study, 95 of 348 (27.3%) participants suffered from the dusk phenomenon. Even in participants with acceptable glycemic control (HbA_1c_ level <7%), there were still 43 of 182 (23.6%) participants with the dusk phenomenon.

Another important finding for our study was that the dusk phenomenon could not only cause hyperglycemia before dinner, but also affect blood glucose in different degrees throughout the day, including nocturnal nadir, fasting, post-breakfast, pre-lunch, and post-dinner glucose (see Fig. 2 and Table 1). The median impacts on HbA_1c_ and 24 hours mean glucose were 0.4 (–0.4, 1.0)% and 0.9 (0.2, 1.4) mmol/L, respectively. Similarly, the dawn phenomenon was reported not only to affect fasting glucose, but also cause the whole blood glucose to increase. The impact of the dawn phenomenon on HbA_1c_ level was about 0.4%, while its impact on averaged 24 hours mean glucose concentrations was about 0.7 mmol/L in diabetic patients with oral antidiabetic agents.^[12]^

No research can explain the mechanism of the dusk phenomenon at present. There is a certain similarity between the dawn phenomenon and the dusk phenomenon in clinical characteristics, from which we may get some enlightenment. The mechanism of the dawn phenomenon is complex and diverse: the loss of β cell function,^[20]^ growth hormone-induced insulin resistance,^[21]^ increasing of circulating insulin-like growth factor binding protein-1 in the morning,^[22]^ poor sleep quality, and the impairment of circadian clock^[23]^ seemed to be closely involved in the pathophysiology of dawn phenomenon in people with diabetes. However, the correlation and regression analysis showed no relationship between the dawn phenomenon and the dusk phenomenon in our study. So we can only speculate on the mechanism of dusk phenomenon. In our study, HOMA-β was significant independent correlates with the dusk phenomenon, which means that loss of β cell function could contribute substantially to the pre-dinner hyperglycemia. In addition, there is a circadian rhythm in human insulin secretion and insulin sensitivity,^[24,25]^ and the circadian rhythm of insulin secretion may be one of the causes of the dusk phenomenon. Furthermore, we speculate that the dusk phenomenon may be related to the weakening of the ambient light during the dinner time. In mammals, the circadian system exists in almost all tissues, including hypothalamic suprachiasmatic nucleus, liver, pancreas, kidney, and leucocytes.^[26]^ The circadian clock are regulated by the 24 hours light and dark cycle produced by Earth's rotation. Disruption of this mechanism could lead to diabetes as a result of a defect in gene expression in the regulation of insulin secretion, and development of pancreatic islets.^[27]^ Sun et al^[28]^ reported an interesting case that a 73-year-old man with bilateral glaucoma (only mild light perception) presented spontaneous and short-term abnormal increase of blood glucose during 9 pm to 3 am. This case may suggest that decreased light reception could affect the circadian rhythm of blood glucose. The mechanism of dusk phenomenon remains to be further studied in pathophysiology.

Postprandial hyperglycemia is a main characteristic of Chinese people with diabetes. A large epidemiological study showed that 46.6% of the people with newly diagnosed type 2 diabetes (44.1% of the men and 50.2% of the women) had isolated increased 2 hours plasma glucose levels in China.^[29]^ So premixed insulin is very suitable and common in Chinese people with diabetes. It had been reported that premixed insulin (twice a day) was prescribed most often in comparison with basal and prandial insulin in Chinese patients treated with insulin.^[30]^ However, premixed insulin twice a day does not seem to be conducive to the control of pre-dinner glucose. Because the dusk phenomenon has a great impact on overall glucose control, we are very concerned about the control of glucose in Chinese diabetic people with dusk phenomenon. We will further study this topic in patients treated with premixed insulin.

There were still limitations in our study that need to be mentioned. First, we had not test the release of growth hormone, which is very important in the pathogenesis of dawn phenomenon in our study. Secondly, although the participants were not allowed to consume any snacks between meals, we still can’t guarantee that we can completely avoid the increase of pre-dinner glucose caused by snacks.

## 5 Conclusions

In conclusion, our study found that: the dusk phenomenon is an objective existence, and the incidence of dusk phenomenon is about 27.3% in people with type 2 diabetes; the impacts of dusk phenomenon on HbA_1c_ and 24-hour mean glucose were about 0.4% and 0.9 mmol/L; there was no relationship between the dawn phenomenon and the dusk phenomenon.

## Author contributions

**Conceptualization:** Yuxin Huang, Xiaoming Tao, Qin Gu, Jiao Sun.

**Data curation:** Yuxin Huang, Yuanyuan Xu, Jieyuzhen Qiu, Cuiping Jiang, Wen Tan, Xiaoming Tao.

**Formal analysis:** Jieyuzhen Qiu.

**Funding acquisition:** Yuxin Huang, Xiaoming Tao, Jiao Sun.

**Investigation:** Yuanyuan Xu.

**Project administration:** Yuxin Huang, Xiaoming Tao.

**Resources:** Cuiping Jiang, Wen Tan, Qin Gu, Jiao Sun.

**Supervision:** Xiaoming Tao.

**Writing – original draft:** Yuxin Huang, Xiaoming Tao.

**Writing – review & editing:** Yuanyuan Xu.
